# The Relationship Between Mental Health and Employment Status Among United States Veterans: A Systematic Review

**DOI:** 10.1093/milmed/usaf452

**Published:** 2025-09-20

**Authors:** Elise V Bailey, Erik J Hansen, Kavita Mosalpuria, Fernando A Wilson

**Affiliations:** Division of Epidemiology, Department of Internal Medicine, School of Medicine, University of Utah, Salt Lake City, UT 84108, United States; Department of Population Health Sciences, School of Medicine, University of Utah, Salt Lake City, UT 84108, United States; Program of Public Affairs, College of Social and Behavioral Science, University of Utah, Salt Lake City, UT 84108, United States; Department of Public Health, Brody School of Medicine, East Carolina University, Greenville, NC 27834, United States; Department of Population Health Sciences, School of Medicine, University of Utah, Salt Lake City, UT 84108, United States; Matheson Center for Health Care Studies, University of Utah, Salt Lake City, UT 84108, United States; Department of Economics, College of Social and Behavioral Sciences, University of Utah, Salt Lake City, UT 84108, United States

**Keywords:** V

## Abstract

**Introduction:**

Post-9/11 U.S. veterans experience disproportionately high rates of mental health conditions. They are also more likely than non-veterans to be unemployed. Many studies have examined potential relationships between those phenomena. We aimed to systematically review this literature.

**Materials and Methods:**

Articles published between September 2001 and September 2021 were identified using PubMed, Web of Science, and Google Scholar. These studies were cohort, cross-sectional, mixed methods, or qualitative studies that reported associations between mental health status or diagnoses and employment and were published in English. Primary mental health conditions of interest were post-traumatic stress disorder (PTSD), alcohol use disorder, bipolar disorder, depression, and other mood, psychiatric, or eating disorders. Two authors screened identified articles for inclusion, and disagreements were resolved by a third author.

**Results:**

Twenty-eight articles met inclusion criteria. Three reported PTSD to be positively associated with unemployment, although 7 found null results. Seven of 9 studies examining depression found positive associations between depression and unemployment, with 2 studies showing null findings. One study reported impaired job performance among veterans with alcohol or substance use disorder, but 2 studies on alcohol use disorder reported no significant association with unemployment. Study methodologies varied significantly, including in their populations, employment definitions, and choice of potential confounders.

**Conclusions:**

The review suggests a relationship between depression and employment status, but evidence for relationships between other conditions and employment status is mixed, which may be because of significant methodological differences between individual studies. Future work should address this by using a generalizable sample of post-9/11 veterans, a standardized definition of unemployment, and base the statistical model on a theoretical framework describing the relationship between mental health and employment.

## INTRODUCTION

There are about 4.7 million post-9/11 U.S. veterans.^1^ Like those who served in other eras, they are more likely than the general population to deal with many health issues, including mental health disorders.^2^ For example, approximately 23% of veterans have post-traumatic stress disorder (PTSD), compared to 3.6% of the general population of U.S. adults.^3^^,^^4^ Additionally, U.S. unemployment trends seem to differ between veterans and non-veterans. Although the actual employment rate among post-9/11 veterans is higher than that in the general population, some literature seems to indicate that they may be more likely to be unemployed than non-veterans with similar characteristics, including sex and race/ethnicity.^5–7^ Although most Veterans successfully transition from the military to civilian employment, a subset of veterans commonly express needing help with the transition to civilian employment.^8^^,^^9^ Perhaps as a result, the VA system has implemented programs to aid veterans in gaining and maintaining employment, including some specifically tailored to veterans with mental health conditions.^10^^,^^11^

In the general population, it is clear that unemployment is higher among individuals with mental illness—including anxiety, depression, PTSD, and bipolar disorder—than among those without, likely because mental health problems negatively impact work entry, retention, attendance, and productivity.^12–18^ If there are also strong relationships between mental illness and unemployment in the post-9/11 veteran population, (1) more research may be needed to understand what may facilitate employment among post-9/11 veterans with mental health conditions and (2) VA employment support programs tailored toward veterans with mental illness should be maintained or perhaps even expanded. Some studies have examined the potential link between mental health disorders and employment status in this population. Our aim is to systematically review this literature, to detect places of consensus and examine limitations that should be addressed in future work.

## METHODS

We searched electronic databases—PubMed, Web of Science, and Google Scholar—using key terms generated and agreed upon by the investigators (see Appendix 1). The use of Google Scholar was intended to identify non-academic research, such as policy reports, for potential inclusion in our review. Our search terms did not include ‘9/11’ or any of the many related synonyms describing military operations post-September 2001. Instead, we conducted searches that included all military-related studies published after September 2001 and manually removed those that did not focus on post-9/11 operations. We screened all PubMed and Web of Science results, and the first 15 pages of Google Scholar results for potentially relevant research. Our initial search was conducted in September 2021. After our initial selection, we screened the references of selected articles for further studies that should be included. No further studies were found.

The included studies met predefined criteria: (1) was a randomized control trial, prospective or retrospective cohort study, cross-sectional study, mixed methods study, or qualitative study; (2) reported associations in the relationship between mental health status or diagnoses and employment among a population of U.S. veterans; (3) was published after September 2001; and (4) was published in English. The primary mental health conditions of interest for our study were PTSD, alcohol use disorder, bipolar disorder, depression, and other mood disorders. However, we also included the terms ‘behavioral health’ and ‘mental health’ in our searches to identify studies examining other conditions. Individuals could have been diagnosed with or screened positive for these conditions at any time relative to their military service.

Studies were included in the review if 2 independent reviewers agreed they met the inclusion criteria. Data were then extracted from the included studies for qualitative review.

## RESULTS

**Figure 1.**
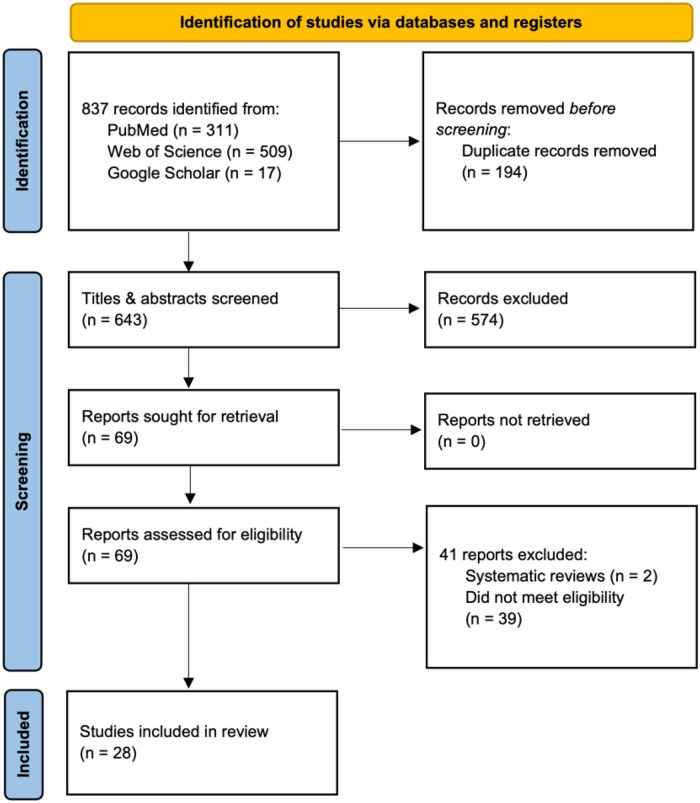
Study flow chart for identification and selection of included studies. Information in this figure was generated by the authors. The template is from the PRISMA (Preferred Reporting Items for Systematic reviews and Meta-Analyses) 2020 reporting guidelines: The PRISMA Group (2021). PRISMA 2020 explanation and elaboration: updated guidance and exemplars for reporting systematic reviews. BMJ 20201; 372: n160. doi: https://doi.org/10.1136/bmj.n160. For more information, visit: http://www.prismastatement.org

Through our search strategy, we identified 837 citations. After 194 duplicate records were removed, 643 articles were screened for title and abstract. After removing irrelevant articles, 69 were retrieved for full text screening. Thirty-Nine of those studies did not meet the eligibility criteria. These were largely studies that included mental health conditions and employment status as potential confounding factors in the literature, but did not present results examining the relationship between them. The association between mental health status or various mental health disorders and employment among veterans was examined in 28 included studies—20 cross-sectional studies, 4 cohort studies, 3 convergent parallel design mixed methods studies, and 1 interview-based qualitative study. A PRISMA diagram depicting the identification process for these articles is shown in **Figure 1**.

Appendix 2 provides details about each study. The most common conditions studied were depression and PTSD. Very few studies examined eating disorders, bipolar disorder, or schizophrenia. Definitions of employment varied somewhat across the studies. Much of the included research had relatively small sample sizes.

### Qualitative Summary of Study Results

#### Outcome: employment status

Twenty studies examined employment status as the outcome variable.

*Depression.* Eight studies found a positive association between depression and unemployment, although 2 studies had null results.^19–28^ One cohort study additionally found that changes in depression status were related to changes in employment status over time—improved depression was associated with increased likelihood of being employed.^29^ A qualitative study found that veterans felt depression impeded their ability to be employed, contributing to becoming homeless.^30^ Finally, 2 studies found that depression was a mediator between other factors and employment status. One found that depression mediated the relationships between some types of traumatic experiences and being out of the workforce, among female veterans.^31^ The other found that depressive symptoms were a mediator in the relationship between physical functioning and employment status.^32^

*Substance use disorder.* Two studies found that substance use was associated with employment status. One found that substance use was positively associated with unemployment among male veterans (results were null among female veterans).^19^ The other found that veterans with a substance use disorder were more likely to be unemployed, disabled, or retired than to be employed.^27^

*Post-traumatic stress disorder.* Three studies found that PTSD was positively associated with unemployment, although 7 found null results.^20–22^^,^^24–27^^,^^29^^,^^32^^,^^33^ In addition, one study found that no PTSD symptom cluster was associated with unemployment.^34^ A qualitative study found that veterans felt PTSD impeded their ability to be employed, which then contributed to becoming homeless.^30^ Lastly, one study found that PTSD mediated the relationships between some types of trauma—including military trauma—and being out of the workforce, among female veterans.^31^

*Anxiety.* Three studies found that anxiety or panic disorders were positively associated with unemployment, although one study had null results.^20^^,^^22^^,^^23^^,^^26^ An additional study found that probable anxiety disorder was associated with job loss.^35^

*Alcohol use disorder.* Two studies examining the relationship between alcohol misuse and employment status had null results.^20^^,^^25^ One found that individuals who screened positive for a probable drinking disorder were more likely to be employed than those who had not.^28^ Another study found that alcohol misuse may be a moderator in the relationship between mental health symptoms and unemployment.^36^

*Eating disorders.* One study examining the relationship between eating disorders and employment status had null results.^37^

*Bipolar disorder.* One study found that veterans with bipolar disorder were more likely to be unemployed, disabled, or retired than to be employed.^27^ A qualitative study found that veterans felt their bipolar disorder impeded their employment, which then contributed to becoming homeless.^30^

*Schizophrenia.* The same study also found that those with schizophrenia were more likely to be unemployed, disabled, or retired than to be employed.^27^

*Presence of any condition.* One study examined the relationship between a variable representing the presence of any mental health condition and unemployment. It found that those with a condition were less likely to be employed than those without.^38^

*Mental health status.* One study found that self-reported mental health status was positively related to unemployment, but another had null results.^20^^,^^39^

*Comorbid conditions.* Three studies examined the relationship between various sets of comorbid mental health conditions and employment status. One found that comorbid depression, mild Traumatic Brain Injury (mTBI), and PTSD was positively associated with unemployment. It also found that comorbid depression and PTSD, without mTBI, was positively associated with unemployment.^40^ The second study found that both comorbid alcohol misuse and depression as well as comorbid alcohol misuse and PTSD were positively associated with unemployment.^36^ The last found that increasing number of mental health diagnoses was positively associated with unemployment.^38^

#### Outcome: mental health status

Two studies examined the relationship between mental health and employment using mental health status as the outcome variable and employment status as the primary predictor of interest. The first found that being employed was significantly associated with a decrease in PTSD severity.^41^ The second found that veterans who were unemployed long-term had more poor mental health days than veterans who were employed and that veterans who were unemployed long-term had more poor mental health days than civilians who were unemployed long-term.^42^

#### Outcome: barriers and facilitators to employment

Four of the located studies included secondary analyses that examined barriers and facilitators to employment among veterans. These incidental findings will be briefly summarized.

*Barriers.* One study compared barriers to employment reported by veterans with PTSD to those reported by veterans with serious mental illness. Veterans with PTSD, both those employed and those unemployed, more commonly listed cognitive symptoms as a barrier to success at work than those with serious mental illness. Unemployed veterans with PTSD reported a lack of work-related skills more frequently than those with serious mental illness. Veterans in both groups commonly reported struggles with interpersonal issues related to their mental health as a barrier.^43^ When employees in VA supportive employment programs were asked to report barriers to employment they feel veterans encounter, psychological stress, mental health, and stigma about mental illness were commonly reported.^44^ In another analysis, Kukla et al. found that mental health and substance use barriers were most commonly reported by veterans who served in combat; however, veterans who did not serve in combat also commonly reported mental illness as a barrier to employment—especially if it was undiagnosed or untreated.^45^ Lastly, veterans with depression or anxiety (examined jointly) reported greater numbers of barriers to employment than veterans without those disorders.^23^

*Facilitators.* In the analysis comparing veterans with serious mental illness to those with PTSD, those with serious mental illness rated vocational assistance as a more significant facilitator to employment than those with PTSD, where those with PTSD said mentorship programs were a facilitator to employment more than those with serious mental illness.^43^ Veterans with PTSD in another study similarly reported social support as a facilitator to employment.^45^

#### Outcome: occupational functioning

Seven of the located studies included secondary analyses that examined occupational functioning outcomes. These incidental findings will, again, be briefly summarized.

*Depression.* One study found that major depressive disorder was associated with impairment in job performance, productivity loss, and productivity costs among employed veterans.^46^ Another found that those who had depression or anxiety (the disorders were examined jointly) was related to lower levels of job search self-efficacy and lower levels of work performance.^23^

*Post-traumatic stress disorder*. Like depression, PTSD was also found in one study to be related to impaired job performance, productivity loss, and productivity costs among employed veterans.^46^ Another found that veterans with PTSD had both lower work-role functioning at baseline and greater declines in work-role functioning over time than veterans without PTSD.^25^ A third found that 4 PTSD symptom clusters—reexperiencing, avoidance, numbing, and hyperarousal—were related to occupational impairment and that all except avoidance were negatively related to occupational satisfaction among female veterans.^34^ The final found that having probable PTSD was related to a significant decrease in overall workforce functioning among male veterans.^33^

*Anxiety.* The study by Adler et al. also examined generalized anxiety disorder and panic disorder. Again, the disorders were negatively associated with impairment in job performance, productivity loss, and productivity costs among employed veterans.^46^ As mentioned previously, those who had depression or anxiety (examined jointly) had lower job search self-efficacy and lower work performance.^23^

*Alcohol and substance use disorder.* Adler et al. also examined alcohol and drug misuse and, as before, found them to be associated with impairment in job performance, productivity loss, and productivity costs among employed veterans.^46^

*Eating disorders.* Although Sienkiewicz et al. found no significant relationship between eating disorder symptoms and unemployment, their results did show a significant negative association between those symptoms and general occupational functioning.^37^

*Any diagnosis or comorbid diagnoses.* The results found by Erbes et al. showed that veterans with any mental health diagnosis or multiple diagnoses had lower work role functioning than those who did not.^25^

### Comparisons

There are important differences between the studies which may contribute to differences in their findings. First, the populations studied varied. Few studied a generalizable sample of post-9/11 U.S. veterans. Some studied VA primary or mental health care patients, others OEF/OIF veterans at post-deployment clinics, others VA patients evaluated for TBI, some female veterans alone, and still others veterans sampled in large surveys such as the Medical Expenditure Panel Survey. Second, although most used valid and reliable measures of mental health diagnoses such as the PTSD CheckList - Military Version (PCL-M) for PTSD and Patient Health Questionnaire-9 (PHQ-9) for depression, the measures used differed. Few considered condition severity. Third, and perhaps most significantly, the definitions of employment categories in the studies varied. For example, some studies defined being employed as encompassing those who were employed full time, employed part time, or students. Others categorized students as unemployed or excluded them altogether. Some studies defined being unemployed as encompassing those who were unemployed for any reason, while others excluded those who were out of the workforce from that definition. Lastly, the studies varied widely in the confounders adjusted for—including physical comorbidities, which are likely to be common in this population. This may be because none of the located studies referenced a specific theoretical framework on which their analysis was predicated. In addition, not all considered the potential impact of symptoms overlapping between conditions. For example, not all studies examining PTSD and employment outcomes adjusted for depression or other conditions whose symptoms overlap with PTSD. As a result, some effect sizes attributed to individual conditions in these studies may be overestimates.

## DISCUSSION

We conducted a systematic review of the literature examining the relationship between mental health status or diagnoses and employment status among post-9/11 veterans. Overall, we see that there may be convincing evidence for a relationship between depression and unemployment among these veterans, which could be related to an underlying association between depression and depressed job performance.

The results for other mental disorders examined in the included literature are more mixed. This may be attributable to differences in study populations, definitions of employment categories, and confounders adjusted for. Other limitations in this literature are a relative dearth of longitudinal studies and the frequent use of relatively small samples. Few of the study samples allow for the study results to reasonably be generalized to the population of all U.S. veterans who served post-9/11, which could very well lead to the mixed results observed in the literature.

Future work should focus on ways to examine a broader population of veterans than, as an example that was common in the literature, VA patients who are known to differ meaningfully from the wider population of veterans.^47^^,^^48^ Future studies should also work to establish a more standardized definition of employment and unemployment in the literature examining those phenomena among veterans. The economics literature frequently utilizes the definition established by the U.S. Bureau for Labor Statistics, which categorizes only those who don’t have employment but are able and willing to work as unemployed.^49^ More frequent use of this or some other definition may yield more consistent results across studies. We believe another potential source of varied results in the literature may stem from serious inconsistencies in the factors adjusted for in the included analyses—including history physical comorbidities and mental health conditions whose symptoms overlap with the condition of interest in the study. This may partly stem from the lack of a specific theoretical framework on which to base the analyses. It is possible that, in future work, models such as social cognitive career theory, economic deprivation models, stress models, control models, or social support models could be used or adapted to form the basis for analyses.^50^^,^^51^ Alternatively, a new model could be developed to describe the relationships between mental health, other predictors, and employment status. Such theoretical work may benefit from considering the possibility that the relationship between mental health and employment may flow in both directions.^52^ The development and use of such a model may facilitate more consistent analytical methods across studies exploring the relationship between mental health and employment among many populations, including U.S. veterans. Further, future work should aim for longitudinal analyses, or analyses using larger sample sizes. Finally, we recommend that all future studies utilize standardized, valid, and reliable measures to detect mental health diagnoses and consider examining both dichotomous variables indicating history of said conditions and their severity.

Our review has limitations. Although our exclusion criteria limited studies to only those published after September 11, 2001, our search terms did not specifically include items to help identify studies that focused on post-9/11 veterans. It is possible that, as a result, some studies in the literature relevant to the research question were omitted. However, our search yielded broader results than the intended criteria and results were manually narrowed to include only articles with a focus on post-9/11 veterans. We believe this makes it unlikely that our review missed any relevant studies because we omitted such terms from our search terms. A further limitation is that we did not exclude any studies on the basis of quality, and the inclusion of lower quality studies may muddle the results. Our findings must be interpreted in light of these limitations.

## CONCLUSION

Our review provides a summary of existing research on the relationship between mental health conditions and employment status among post-9/11 veterans. The evidence suggests a relationship between depression and employment status in this population, which may be related to an underlying relationship between depression and job performance. The evidence for relationships between other conditions and employment status is more mixed, which may be because of significant methodological differences between individual studies. Future work should address this by using a generalizable sample of post-9/11 veterans, a standardized definition of unemployment, and base statistical models on a theoretical framework describing the relationship between mental health and employment. Future work should also examine on less commonly researched conditions such as bipolar disorder and schizophrenia.

## Supplementary Material

usaf452_Supplementary_Data

## Data Availability

All data are publicly accessible.

## INSTITUTIONAL CLEARANCE

Institutional clearance does not apply.
