# Supplementary material for: The Relationship Between Mental Health and Employment Status Among United States Veterans: A Systematic Review
Source: Mil Med. 2025 Sep 20;191(3-4):e571–7. doi: 10.1093/milmed/usaf452 (PMC13017545; doi:10.1093/milmed/usaf452)
Supplement: usaf452_Supplementary_Data [file usaf452_supplementary_data.zip › MH Systematic Review Appendix 1.docx]

APPENDIX 1: Database Search Terms

**PubMed search terms:**

((TMD[Title/Abstract]) OR (TMJ[Title/Abstract]) OR (PTSD[Title/Abstract]) OR (alcohol abuse[Title/Abstract]) OR (alcoholism[Title/Abstract]) OR (bipolar[Title/Abstract]) OR (depression[Title/Abstract]) OR (mood disorder[Title/Abstract]) OR (behavioral health[Title/Abstract]) OR (mental health[Title/Abstract])) AND ((employment[Title/Abstract]) OR (unemployment[Title/Abstract])) AND ((veterans[Title/Abstract]) OR (military[Title/Abstract]) OR (service members[Title/Abstract]))

**Web of Science search terms:**

(AB=(mental) OR AB=(behavioral) OR AB=(TMD) OR AB=(TMJ) OR AB=(PTSD) OR AB=(alcohol abuse) OR AB=(alcoholism) OR AB=(bipolar) OR AB=(depression) OR AB=(mood disorder)) AND (AB=(veteran) OR AB=(military) OR AB=(service member)) AND (AB=(employment) OR AB=(unemployment))

**Google Scholar search terms:**

veteran employment mental OR behavioral OR TMD OR TMJ OR PTSD OR alcohol abuse OR alcoholism OR bipolar OR depression OR mood OR unemployment OR military OR service member
