# Supplementary material for: The Relationship Between Mental Health and Employment Status Among United States Veterans: A Systematic Review
Source: Mil Med. 2025 Sep 20;191(3-4):e571–7. doi: 10.1093/milmed/usaf452 (PMC13017545; doi:10.1093/milmed/usaf452)
Supplement: usaf452_Supplementary_Data [file usaf452_supplementary_data.zip › MH Systematic Review Appendix 2 - clean.docx]

| **Appendix 2. Study characteristics** | | | | | | |
| --- | --- | --- | --- | --- | --- | --- |
| **Study** | **Study Design** | **Sample (n)** | **Duration** | **Sample Characteristics** | **Results** | **Strengths/Limitations** |
| Adler et al. (46) | Cross-sectional | 473 | N/A | OEF-OIF veterans referred by PCP for MH assessment; interviewed between 08/2007 and 04/2009; patients in VA medical centers located in Pennsylvania, Maryland, and Upstate New York | MDD was associated with impairment in mental-interpersonal demands (β = .28), time management (β = 0.15), and output (β = 0.13), productivity loss (β = 0.21), and an annual productivity cost of $929. PTSD was associated with impaired work performance in mental-interpersonal demands (β = 0.16), time management (β = 0.14), and output (β = 0.12), as well as productivity loss (β = 0.16) and an annual productivity cost of $651. GAD/panic disorder was associated with work impairment in terms of mental-interpersonal demands (β = 0.20), time management (β = 0.26), and output (β = 0.16), as well as productivity loss (β = 0.23) and an annual productivity loss of $904.Alcohol dependence and drug abuse were associated with impairment in job performance in terms of output (β = 0.12) and physical demands (β = 0.13), as well as productivity loss (β = 0.11), and annual productivity costs of $561. | Strengths: valid measures of primary outcomes and primary predictors of interest;  Limitations: relatively small sample size, limited generalizability |
| Amara et al. (19) | Cross-sectional | 63,795 | N/A | Post-9/11 veterans who completed VA comprehensive TBI evaluation between 07/2009 and 09/2013 | Among male veterans, depression and drug abuse positively associated with unemployment (aOR = 1.13 and 1.28, respectively); among female veterans, no condition examined was significantly associated with unemployment | Strengths: a large proportion of the study sample was suspected to have a psychiatric condition;  Limitations: does not adjust for race; limited generalizability, relatively small number of female veterans in each employment category, assessment of psychiatric conditions is not standardized and is left to clinician impression |
| Amick et al. (40) | Cross-sectional | 48,821 | N/A | OEF/OIF veterans who had TBI evaluation in any VA between the end of 2009 and end of 2013 | Those with all three conditions and those with both PTSD + depression had increased risk of unemployment (RR = 1.29-1.73 and 1.28-1.67, respectively) | Strengths: large sample;  Limitations: limited generalizability, assessment of psychiatric conditions is not standardized and is left to clinician impression, did not adjust for sex or race or physical conditions which might cause workforce exit |
| Burnett-Ziegler et al. (20) | Cross-sectional | 585 | N/A | OEF/OIF National Guard veterans in the Midwest U.S. who attended mandatory Reintegration Workshops 45-60 days after demobilization. All were demobilized between 02/2009 and 09/2009. | Among those employed, those with better mental health status were more likely to be employed full-time (OR = 1.047) | Strengths: adjusts for number of deployments and combat exposure;  Limitations: small sample size, 45-60 days post-deactivation may be a very short period in which to find a job during the Great Recession, limited generalizability |
| Cohen et al. (24) | Cross-sectional | 169 | N/A | OEF/OIF veterans who visited a post-deployment clinic in Boston and who had completed TBI evaluation between 12/2009 and 05/2010 | Depression was related to employment status (aOR = 0.21). PTSD was not. | Strengths: unemployment was high in the sample, morbidity may also have been high in the sample;  Limitations: regression does not adjust for race or any SES variable, very limited generalizability, very small sample size |
| Erbes et al. (25) | Cohort | 262 | 1 year | Army National Guard Reserve members deployed to OIF between 03/2006 and 07/2007 who enrolled and completed interviews within a year of return | No diagnosis (PTSD, MDD, alcohol misuse) was significantly related to employment status; those with several diagnoses had lower work role functioning (β for subthreshold PTSD = -0.14, β for MDD = 0.16, β for alcohol misuse = 0.20); those with PTSD showed greater declines in work role functioning over time (β = 0.19). | Strengths: measurement at multiple times;  Limitations: no adjustment for any variables in the relationship between diagnoses and employment, very small sample size, limited generalizability |
| Goetz et al. (38) | Cross-sectional | 1,047 | N/A | Veterans with SCI receiving care for that injury in various VA SCI centers who participated in PrOMOTE between 8/2010 and 3/2015 | No individual mental health diagnosis was significantly related to employment; increasing number mental health diagnoses was negatively associated with employment (aOR = 0.46 per 2 unit increase) | Strengths: relatively large sample size, may be nationally representative of veterans with SCI; Limitations: not generalizable beyond those with SCI |
| Hamilton et al. (21) | Cross-sectional | 1,605 | N/A | Women veterans who served between 2008-2009 | Screening positively for depression was significantly related to unemployment (aOR = 4.7); no significant relationship between PTSD and unemployment | Strengths: relatively large sample, nationally representative of women veterans;  Limitations: survey conducted only by telephone, everything was self-reported, unable to consider whether participants had children |
| Horton et al. (22) | Cohort | 9,099 | 3-6 years | Panels 1 and 2 of regular active duty, Reserve, and National Guard members sampled as part of the Millenium Cohort Study, enrolled between 2001 and 2006, who were active duty at time of enrollment but separated between enrollment and follow-up | PTSD was not associated with unemployment, but depression and anxiety/panic disorder were--although only among those with routine retirement (aOR = 1.67 and 1.63, respectively. | Strengths: large sample, long follow-up, adjusted for military-related confounders, cohort representative of U.S. military;  Limitations: couldn't consider reasons for unemployment such as being in school, potential issues caused by attrition, everything was self-reported, did not adjust for severity of disease |
| Kintzle et al. (36) | Cross-sectional | 126 | N/A | Enrolled from specific deployed NG unit in 08/2011 at the unit's postdeployment decompression site | Those with comorbid alcohol misuse and depression or comorbid alcohol misuse and PTSD were less likely than those without comorbidity to be employed (p from Chi square tests = 0.01 and -.02, respectively; alcohol misuse may moderate the relationship between mental health symptoms and unemployment—the interaction of unemployment and alcohol misuse was significantly related to depression (aOR = 6.97), and to PTSD (aOR = 12.99). | Strengths: ;  Limitations: very small convenience sample, not able to adjust for race and other potential confounders such as physical conditions |
| Krull and Oguz (28) | Cross-sectional | 9,125 | N/A | Alumni of the WWP, who would have been veterans injured in combat post-9/11 | Those with self-reported depression, probable depression, and other severe mental injuries were less likely to be employed than those without (aOR = 0.85, 0.67, and 0.75, respectively). Those with probable problem drinking were more likely to be employed than those without it (aOR = 1.19). | Strengths: large sample; Limitations: may not be generalizable to veterans not injured in combat |
| Kukla et al. (2015, Factors) (43) | Convergent parallel mixed methods | 40 | N/A | Veterans receiving VA care in a single Midwestern city, from outpatient clinics for mental health disorders such as PTSD, SMI, and general outpatient mental health care--must have had PTSD or SMI | Unemployed people with PTSD rated lack of work skills as a more significant barrier to work success then those with SMI; those with SMI rated vocational assistance as a more significant facilitator to work success than those with PTSD; individuals with PTSD often discussed cognitive symptoms as barriers to work success, where those with SMI generally did not; those with PTSD (both employed and unemployed) commonly expressed that veteran-centered mentorship programs facilitated their transition to civilian life; veterans in all groups said they struggled with interpersonal issues with coworkers related to their mental health issues | Strengths: inclusion of veterans with PTSD which was rarely done at the time;  Limitations: small sample, unknown reliability and validity of survey |
| Kukla et al. (2016) (44) | Convergent parallel mixed methods | 114 | N/A | VA employees involved in IPS SE programs, nationwide, between 11/2013 and 12/2013 | Psychological stress and mental health were listed among the most common highly rated barriers to work success; SE employees also noted that stigma about mental illness among employers was a barrier to work success for veterans with mental illness | Strengths: perspective of people who help multiple veterans find and maintain employment;  Limitations: unknown reliability and validity of survey, may not be generalizable--especially because providers helped few veterans with PTSD and mostly those with SMI |
| Kukla et al. (2015, Mixed) (45) | Convergent parallel mixed methods | 40 | N/A | Veterans receiving VA mental health care at a VAMC in a single midwestern city--specifically three outpatient clinics specializing in PTSD, SMI, and general outpatient mental health care--must have had PTSD or SMI (bipolar, MDD, or schizophrenia spectrum disorder) | Veterans who served in combat more frequently noted mental health and substance use as barriers to finding and keeping jobs; veterans who did not serve in combat also noted mental health as a barrier to finding and keeping work--in particular, undiagnosed or untreated mental illness; participants discussed social support as being an important facilitator to finding and keeping jobs when they had PTSD | Strengths: able to compare experiences of combatants and non-combatants;  Limitations: small sample, unknown reliability and validity of survey, may not be generalizable, recall bias |
| Metraux et al. (30) | Qualitative (interview-based) | 17 | N/A | Male adult veterans who separated after 08/2001 and were homeless in metropolitan Philadelphia and southern New Jersey, with interviews between 3/2014 and 2/2016 | Veterans identified mental illnesses such as PTSD, MDD, and bipolar disorder as impeding employment, which then contributed to becoming homeless | Strengths: first to apply qualitative methods to the question in the population of male veterans;  Limitations: very small sample, likely not generalizable, recall bias |
| Pogoda et al. (26) | Cross-sectional | 11,683 | N/A | OEF/OIF veterans who had TBI evaluation in any VA between 10/2007 and 06/2009 | Among veterans with no TBI, depression and anxiety were associated with being unemployed and looking for work (aOR = 1.26 and 1.39, respectively). Among those with moderate/severe TBI, PTSD and anxiety were associated with being unemployed and looking for work (aOR = 1.52 and 1.41, respectively). | Strengths: very large sample;  Limitations: assessment of psychiatric conditions is not standardized and is left to clinician impression, may not be generalizable to veterans not evaluated for TBI, couldn't adjust for race or other potentially meaningful confounders |
| Possemato et al. (41) | Cross-sectional | 150 | N/A | Combat-exposed veterans with PTSD and hazardous alcohol use in NY and Tennessee, who were deployed to OEF/OIF within previous five years | Employment status was a significant predictor of PTSD severity (β = -0.15) | Strengths: valid and reliable measure of PTSD severity;  Limitations: small sample, unclear definitions of employment, inclusion criteria applied differently in different sites, didn't adjust for race or other potentially meaningful confounders |
| Schnurr and Lunney (34) | Cross-sectional | 253 | N/A | Female veterans and active duty personnel from an RCT for PTSD treatment drawn from 9 VA centers, 2 VA readjustment centers, and 1 military hospital between 2002 and 2005--must have had PTSD with severity of at least 45 on CAPS whose traumatic event was experienced at least 3 months prior, had clear memory of the trauma, did not receive outside psychotherapy for PTSD during the trial, and stable drug regimen for at least 2 months | No PTSD symptom cluster was related to employment status, but all clusters were related to occupational impairment even when adjusting for depression (β for reexperiencing, avoidance, numbing, and hyperarousal were 0.29, 0.26, 0.20, and 0.27, respectively).All except avoidance were related to occupational satisfaction, when not adjusting for depression (β for rexperiencing, numbing, hyperarousal, and depression symptom clusters were -0.18, -0.18, -0.21, and -0.30, respectively). | Strengths: examined PTSD in a more detailed way than binary measure, used valid measure of PTSD symptoms, able to examine women;  Limitations: small sample, did not adjust for many potential confounders, may not be representative of all women veterans |
| Sienkiewicz et al. (2020) (31) | Cohort | 369 | 1 year | Female veterans in VA data warehouse, contacted in 2012 and 2013 for larger study on IPV | No trauma types were associated with employment status; PTSD and depression both mediated relationships between some trauma types and being out of the workforce. For example, both depression and PTSD symptom scores mediated the relationship between military-related trauma and being out of the workforce. This study had extensive significant findings. Please refer to it for other significant findings. | Strengths: able to examine women veterans;  Limitations: small sample size, only adjusted for age and sex and education, may not be generalizable, mailed survey may exclude homeless veterans |
| Sienkiewicz et al. (2021) (37) | Cross-sectional | 198 | N/A | Female veterans in VA data warehouse, contacted in 2012 and 2013 for larger study on IPV | ED symptoms were significantly negatively associated with occupational functioning (β = -0.25); no relationship between ED symptoms and unemployment or being out of the workforce; depressive symptoms was a mediator between ED symptoms and occupational functioning. The unstandardized coefficient for the indirect effect of depressive symptoms in the relationship between eating disorder symptoms and occupational functioning was -0.38. | Strengths: able to measure ED symptoms;  Limitations: could not confirm ED diagnoses, few unemployed individuals in sample, small overall sample size, very few individuals with probable ED, sample was mostly white, did not adjust for many potential confounders |
| Smith et al. (2015) (39) | Cross-sectional | 19,600 | N/A | Families sampled in the 2010 MEPS | Self-reported mental health status was not significantly related to employment among veterans. | Strengths: large sample size that's nationally representative;  Limitations: no details about other factors related to history as a veteran such as length of active duty status, everything was self-reported and not according to highly valid/reliable measurements |
| Tran et al. (42) | Cross-sectional | 47,867 | N/A | People sampled in the 2012 BRFSS | Unemployed veterans (long-term) had more poor mental health days than veterans who were employed (reference group was currently employed civilians; β for employed veterans = 1.3 with 95% CI: 0.54-2.05 and β for long-term unemployed veterans = 7.45 with 95% CI: 4.57-10.34). They also had more poor mental health days than civilians who were unemployed long-term (β for long-term unemployed civilians = 3.33 with 95% CI: 2.50-4.16). | Strengths: large sample size, nationally representative;  Limitations: unable to control for factors related to military service |
| Umucu et al. (35) | Cross-sectional | 6,607 | N/A | Participants recruited from Amazon Mechanical Turk who were at least 22 years old, U.S. residents, and made $75,000 or less in income per year. They also had to be highly active on the site. | Among veterans, probable anxiety was significantly related to job loss (aOR = 2.95), although probable depression, probable COVID-19 related stress, and loneliness were not.The OR for the relationship between probable anxiety and job loss was much larger for veterans than for civilians (aOR of 2.95 vs. aOR = 1.48). | Strengths: large sample size, including relatively large group of veterans;  Limitations: sample is likely not nationally representative, likely omitted confounding variables such as factors related to military status and mental health and factors related to job loss; job loss was self-reported and may be biased |
| Vogt et al. (33) | Cross-sectional | 524 | N/A | Sample of post-9/11 veterans from DoD database who separated from service between 2008 and 2010 and were surveyed multiple times | Having probable PTSD appeared related to significant impairment in workforce functioning for men (β = 9.73) but was not significantly related to unemployment or being out of the workforce in either male or female veterans | Strengths: able to adjust for military-related factors, used valid measurements for many variables;  Limitations: relatively small sample that may not be generalizable |
| Winter et al. (32) | Cross-sectional | 83 | N/A | Veterans with TBI from a single VA's Rehabilitation Medicine Service, from any conflict from the Vietnam War to the present, who speaks English, and has a family member living either with them or near by | PTSD was not significantly associated with employment status, but depressive symptoms was found to be a mediator in the relationship between physical functioning and employment status (The addition of depressive symptoms to regression modeling resulted in physical functioning variables becoming insignificant in the model; aOR for depressive symptoms = 0.875). | Strengths: Considered different exposure levels;  Limitations: almost all of the sample was male, small sample size |
| Zivin et al. (2011) (27) | Cross-sectional | 98,867 | N/A | Veterans sampled in the Survey of Healthcare Experiences of Patients in 2005 who had valid employment data and were of working age | Those who were unemployed were more likely to have withschizophreniaor a SUD than those who were employed (OR = 2.2 for both).Those who were disabled were more likely to have schizophrenia, PTSD, bipolar disorder, and depression than those who were not disabled (OR = 5.6, 2.5, 2.2, and 2.0, respectively) Those who were retired were more likely to have schizophrenia or PTSD than those who were not retired (OR = 3.4 and 1.5, respectively). | Strengths: large sample size in population of veterans with high risk for unemployment;  Limitations: probably has limited representativeness for post-9/11 veterans given survey date, may not be representative of all veterans since most do not receive VA health care |
| Zivin et al. (2012) (29) | Cohort | 516 | 18 months | Working age veterans receiving primary care for a mental health disorder in a VA center in 5 states who received primary care at least once in the past 12 months and had another visit scheduled within the next 3 months following selection; had to have depression | Changes in depression status are related to changes in employment status over time--improved depression was associated with increased likelihood of being newly employed ARR = 6.7). PTSD was also related to lower likelihood of consistent employment (ARR = 0.45). | Strengths: longitudinal, able to adjust for severity, able to measure exposure and outcome multiple times;  Limitations: likely not generalizable outside VA patients, some issues with small cell sizes |
| Zivin et al. (2016) (23) | Cross-sectional | 287 | N/A | Working-age veterans receiving VA primary care in a Midwestern VA center between 6/2014 and 7/2014 | Depression/anxiety is related to lower likelihood of being employed (aOR = 0.49), lower levels of job search self-efficacy (β = -0.23), lower levels of work performance (β = -0.305), and greater numbers of barriers to employment (β = 0.305) | Strengths: able to examine social support and military-related factors;  Limitations: small cell sizes, may not be generalizable beyond the center at which the survey was done, examined only depression and anxiety combined |
